# Tranexamic Acid in the Management of Hemoptysis: Pharmacology, Administration Routes, Clinical Evidence, and Practical Considerations

**DOI:** 10.7759/cureus.108790

**Published:** 2026-05-13

**Authors:** Stephanie R Schmalzried, Muneebah Munir, Bharat Bajantri

**Affiliations:** 1 Pharmacology, Parkview Health, Fort Wayne, USA; 2 Internal Medicine, Parkview Health, Fort Wayne, USA; 3 Pulmonary and Critical Care Medicine, Parkview Health, Fort Wayne, USA

**Keywords:** antifibrinolytic therapy, critical care, hemoptysis, pulmonary hemorrhage, tranexamic acid

## Abstract

Hemoptysis is a frequent and potentially life-threatening pulmonary presentation that requires rapid stabilization and timely definitive management. Tranexamic acid (TXA), a synthetic antifibrinolytic agent, has gained increasing interest as an adjunctive medical therapy for hemoptysis, supported by recent randomized trials, observational studies, and systematic reviews evaluating inhalational and endobronchial routes. While bronchoscopy, bronchial artery embolization (BAE), and surgery remain central to the management of severe or life-threatening hemoptysis, TXA may reduce bleeding burden, shorten hospitalization, and serve as a temporizing or bridging therapy while definitive interventions are arranged. This narrative review summarizes TXA pharmacology, available routes of administration, dosing strategies, clinical evidence, safety considerations, and practical integration into contemporary hemoptysis management pathways. The literature selection approach is briefly described in the manuscript body to improve transparency regarding how representative studies were identified and prioritized.

## Introduction and background

Hemoptysis encompasses a broad clinical spectrum, ranging from blood-streaked sputum to massive or life-threatening hemorrhage capable of causing airway obstruction, gas exchange impairment, hemodynamic instability, or cardiovascular collapse [1]. Definitions of severe or massive hemoptysis vary across studies, with some using volume-based thresholds and others emphasizing clinical consequences such as airway compromise or need for urgent intervention. Reported mortality in severe or life-threatening hemoptysis ranges from 30% to over 50%, depending on bleeding severity, underlying etiology, comorbidities, and treatment setting [1-3]. Effective management relies on rapid airway protection, prompt localization of bleeding, and escalation to appropriate diagnostic and therapeutic interventions [1,2]. Bronchoscopy plays an important diagnostic and therapeutic role, while bronchial artery embolization is often favored as a definitive first-line intervention for massive or life-threatening hemoptysis when available; surgery is generally reserved for selected cases or when less invasive measures fail. Medical therapies may play an important adjunctive role, particularly when bleeding is nonmassive, when definitive procedures are being arranged, or when procedural intervention is not immediately available or clinically appropriate. In this context, tranexamic acid (TXA) may help address an important management gap by serving as a temporizing or bridging therapy to reduce bleeding burden while diagnostic evaluation and definitive interventions proceed [4,5]. The TXA has gained increasing interest for hemoptysis, supported by emerging evidence from randomized trials, observational studies, and recent systematic reviews; however, available data remain limited by small sample sizes, heterogeneous patient populations, variable definitions of hemoptysis severity, and inconsistent dosing strategies across intravenous (IV), inhalational, and endobronchial routes [4-7]. 

## Review

Literature selection 

For this narrative review, relevant literature was identified through searches on Google Scholar, journal databases, clinical case report sources, and citation chaining from known articles. Search terms included “tranexamic acid,” “TXA,” “hemoptysis,” “pulmonary hemorrhage,” “nebulized,” “inhaled,” “endobronchial,” and “bronchoscopy.” No formal date restriction was applied because literature for some indications and administration routes remains sparse; however, more recent studies were prioritized when available, particularly when they added data not included in previously published reviews. Randomized trials, observational studies, systematic reviews, case series, and case reports were considered for inclusion. Studies were prioritized if they evaluated TXA use for hemoptysis, pulmonary hemorrhage, or bronchoscopic bleeding control and reported clinically relevant information on route of administration, dosing, indication, outcomes, or adverse events. Animal studies, non-English publications, and studies not involving pulmonary bleeding were excluded. All authors contributed to study identification, duplicate removal, and review of candidate studies in a shared spreadsheet. Because this was a narrative review rather than a systematic review, no formal meta-analysis, meta-regression, pooled statistical analysis, or formal risk-of-bias assessment was performed. Quantitative findings, including p-values and effect estimates, were extracted from the original studies when reported and are presented descriptively to provide clinical context rather than to infer pooled efficacy. Study design, sample size, comparator use, outcome definitions, route of administration, dosing variability, and adverse-event reporting were considered when interpreting the strength and limitations of the evidence. 

Pharmacology and mechanism of action 

Tranexamic acid, a synthetic lysine analog, inhibits fibrinolysis by reversibly blocking lysine-binding sites on plasminogen, thereby reducing plasmin formation and limiting fibrin clot degradation [7,8]. This primary antifibrinolytic effect helps preserve clot stability and may reduce ongoing airway bleeding. Additional effects on kallikrein activation and inflammatory mediator pathways have been described, but their clinical relevance in hemoptysis remains uncertain and should be considered less well established [7,8]. 

Routes of administration and dosing 

Tranexamic acid can be administered through several routes, including IV, oral, and inhalation delivery [5-7,9-11]. Intravenous administration provides rapid systemic exposure with high circulating drug levels, supporting its use when immediate control of bleeding is necessary [9,10]. Oral TXA results in slower and incomplete absorption, leading to lower bioavailability and delayed attainment of therapeutic levels [9]. Inhaled TXA has emerged as an off-label route of interest for localized management of pulmonary bleeding; although pharmacokinetic data remain limited, clinical reports suggest rapid hemostatic activity within the airways [9]. Variability in dosing strategies, delivery methods (nebulized vs. endotracheal instillation), and absence of standardized protocols continue to limit widespread adoption of inhaled TXA. 

Intravenous TXA 

Intravenous TXA dosing in hemoptysis studies has varied across protocols rather than following a standardized escalation strategy [12-17]. Commonly reported regimens include 500 mg to 1,000 mg IV every six to eight hours, with some studies using higher total daily doses or cumulative doses up to approximately 2,000 mg or more, depending on bleeding severity, treatment duration, and institutional practice [12-16]. Because optimal dosing has not been established, IV TXA should be individualized based on the severity of bleeding, renal function, thrombotic risk, and local protocol. 

Clinical evidence supporting IV TXA remains variable. While some studies demonstrate reductions in bleeding volume or decreased need for procedural intervention, other studies and pooled analyses have not consistently demonstrated significant improvement across key outcomes, including bleeding duration, recurrence, or need for invasive intervention [4,12-16]. Limited comparative data have evaluated nebulized versus IV TXA, and available findings should be considered preliminary. Some studies suggest potential advantages of nebulized administration in selected nonmassive or localized hemoptysis, but the evidence is not sufficiently robust to establish superiority over IV therapy [13]. Observational data from a nationwide propensity-matched cohort of critically ill patients found that TXA use was associated with shorter hospital stays, lower in-hospital mortality, and reduced healthcare costs; however, these findings should be interpreted as associations rather than evidence of causal benefit or dose-dependent efficacy [14]. The observed relationship with lower cumulative doses likely reflects differences in patient selection, bleeding severity, treatment duration, or clinical practice patterns rather than a defined optimal dosing threshold. Additionally, pooled data from randomized trials have shown a reduction in bleeding duration compared to placebo, though the overall quality of evidence remains limited [5,12]. 

Despite these potential benefits, systemic administration carries a risk of thromboembolic complications, particularly among patients with malignancy or underlying prothrombotic conditions [10]. Careful patient selection and individualized risk assessment remain important considerations when initiating IV TXA therapy.

 *Oral TXA *

There is currently insufficient evidence to recommend oral antifibrinolytics for pulmonary hemorrhage. Only one randomized controlled trial was identified using oral TXA for hemoptysis [17]. In that study, TXA 500 mg orally every eight hours for up to seven days did not significantly shorten the duration of hemoptysis compared with placebo, and baseline imbalances between groups limited interpretation [17]. Given its slower absorption, lower systemic bioavailability, and limited hemoptysis-specific evidence, oral TXA may be a pragmatic consideration in selected cases of mild or recurrent hemoptysis when acute bleeding control is not required. However, this should not be interpreted as an evidence-based recommendation, and further studies are needed to define its role. 

*Inhalational (Nebulized) TXA* 

Nebulized TXA delivers high local drug concentrations to the airway with minimal systemic absorption [9,11]. Reported dosing regimens in the literature typically range from 250 mg to 500 mg diluted in 5 mL normal saline, administered every six to eight hours for 24 to 72 hours, with some cases extending use up to five days [6,13,18-21]. A randomized, double-blind, placebo-controlled trial demonstrated significantly faster resolution of hemoptysis among patients treated with nebulized TXA, with most achieving bleeding cessation within five days [6]. Subsequent randomized trials and observational studies have similarly shown reduction in bleeding frequency and volume, along with a decreased need for bronchoscopic or angiographic intervention [18-20]. A 2025 systematic review and meta-analysis reported that inhalational TXA was associated with a higher likelihood of hemoptysis cessation and reduced need for invasive procedures in nonmassive hemoptysis; however, absolute cessation rates and effect sizes varied across included studies because of differences in patient populations, bleeding severity, dosing regimens, and outcome definitions [21]. 

*Endobronchial TXA * 

Endobronchial administration of TXA involves delivering the medication directly to the site of bleeding through a bronchoscope. Reported dosing ranges from 250 mg to 1,000 mg, instilled locally to achieve targeted hemostasis [22-25]. This route has been described as an adjunctive measure during bronchoscopy for focal airway bleeding, malignancy-related hemoptysis, and post-procedural hemorrhage [22,25]. Evidence supporting endobronchial TXA remains limited to small case series and reports, but existing observations suggest it may be a useful option when bleeding is localized and accessible during bronchoscopic intervention [22-25]. 

Combined Systemic and Inhalational Therapy 

Case reports and small observational series describe the use of combined IV and nebulized TXA for moderate to severe hemoptysis, demonstrating rapid stabilization without significant adverse effects [26]. Although these findings are promising, evidence remains limited, and controlled trials evaluating therapy are currently lacking. 

Contraindications 

Tranexamic acid is well tolerated across administration routes; however, careful patient selection remains essential to minimize adverse outcomes. Absolute contraindications include active intravascular thrombosis and known hypersensitivity to TXA, as inhibition of fibrinolysis may worsen existing thrombosis or precipitate severe reactions in sensitized individuals [8-10]. Relative contraindications require individualized clinical judgment and include a history of venous or arterial thromboembolism, severe renal impairment, and underlying prothrombotic conditions. Because TXA is eliminated primarily unchanged by the kidneys, patients with moderate-to-severe renal dysfunction require dose adjustments when receiving IV formulations to avoid drug accumulation and excessive systemic exposure [9,10]. Patients with prior thromboembolic disease or inherent clotting disorders may be at heightened risk, as reduced fibrinolysis may promote clot stability in a manner that exacerbates underlying baseline thrombotic risk. 

Although systemic TXA carries a theoretical risk of thromboembolic complications through inhibition of fibrinolysis, clinically observed thrombotic events have been uncommon in available hemoptysis studies. However, these studies are generally limited by small sample sizes, short follow-up, and inconsistent adverse-event reporting, which restricts conclusions about true thrombotic risk. Inhalational administration may result in lower systemic exposure and therefore may theoretically reduce thrombotic risk compared with IV therapy, but definitive comparative safety data are lacking [13,19-22]. Clinicians should therefore distinguish mechanistic concern from observed clinical evidence and apply individualized risk assessment, particularly in patients with prior thrombosis, malignancy, renal impairment, or other prothrombotic conditions. 

Adverse effects 

Available evidence suggests that TXA has a generally favorable safety profile when used for pulmonary hemorrhage, although hemoptysis-specific safety data remain limited. Systemic TXA may cause gastrointestinal symptoms, infusion-related hypotension, rare seizure activity, visual disturbances including chromatopsia, and thromboembolic complications [7-10]. Across broader clinical use, thromboembolic events have been reported infrequently with TXA, although risk may be higher in patients with active thrombosis, prior thromboembolic disease, malignancy, renal impairment, or other prothrombotic conditions [10]. In hemoptysis, a Cochrane review of two randomized controlled trials did not identify adverse effects clearly attributable to TXA’s antifibrinolytic mechanism and found no significant difference in mild side effects between TXA and placebo groups [12]. However, the included studies were small and not designed to reliably detect rare, delayed, or thromboembolic adverse events; therefore, the absence of a strong safety signal should not be interpreted as evidence that these risks are absent. 

Nebulized TXA appears to be well tolerated in available studies. Reported adverse effects include mild cough, throat irritation, and rare bronchospasm, typically responsive to bronchodilators. Randomized trials and meta-analyses have not demonstrated an increase in serious adverse events with nebulized TXA, but available safety data remain limited by small sample sizes, short follow-up, and inconsistent adverse event reporting [11,18-21].

Clinical indications 

Tranexamic acid may be considered in select clinical contexts, including non-massive hemoptysis, bronchiectasis-associated bleeding, malignancy-related hemoptysis, infection-associated bleeding, and as bridging therapy while definitive interventions are being arranged. Table 1 summarizes representative key studies identified through the literature selection process described above, with emphasis on route of administration, dosing strategy, clinical indication, outcomes, and adverse events. Overall, the studies summarized in Table 1 demonstrate substantial heterogeneity in study design, patient populations, hemoptysis severity, underlying etiology, route of TXA administration, dosing strategy, and outcome definitions. Statistical findings are reported descriptively as presented in the original studies and should be interpreted cautiously because of heterogeneity in study design, small sample sizes, and inconsistent outcome definitions. The strongest evidence comes from small randomized controlled trials evaluating nebulized or IV TXA, while much of the evidence for endobronchial, oral, combined-route, malignancy-related, and diffuse alveolar hemorrhage use remains limited to observational studies, case series, or case reports. Importantly, Table 1 also includes studies and pooled analyses that did not demonstrate significant improvement across all measured outcomes, underscoring the mixed nature of the evidence base and reducing the risk of selective presentation bias. Several studies rely on short-term outcomes such as bleeding cessation, reduction in bleeding volume, or need for invasive intervention, and most are underpowered to assess recurrence, mortality, thromboembolic risk, or other rare adverse events. Therefore, Table 1 should be interpreted as a balanced synthesis of available clinical evidence rather than proof of uniform efficacy across all routes, etiologies, or severities of pulmonary hemorrhage. 

**Table 1:** Studies of TXA in pulmonary hemorrhage COPD: Chronic obstructive pulmonary disease; TXA: Tranexamic acid

Study	Study design	Patients	Diseases	Intervention	Outcomes	Adverse events
Wand et al., 2018 [6]	Prospective, double-blind, randomized controlled trial	47 patients (TXA group: 25, placebo group: 22)	Hemoptysis in COPD, bronchiectasis, and malignancy	Nebulized TXA 500 mg every eight hours vs. placebo (normal saline)	The TXA group experienced a significant reduction in hemoptysis volume, a higher resolution rate within five days (96% vs. 50%; p<0.005), needed fewer invasive procedures, and experienced a significantly lower recurrence rate at one year.	No adverse effects were reported in the study.
Prutsky et al., 2016 [12]	Cochrane systematic review	Two randomized controlled trials; 70 total patients	Hemoptysis from mixed etiologies, including tuberculosis	Oral or IV TXA compared with placebo or no treatment	TXA reduced bleeding time compared with placebo or no treatment (weighted mean difference, -19.47 hours; 95% confidence interval, -26.90 to -12.03; I²=52%) but did not significantly improve hemoptysis remission at seven days.	No severe adverse effects were reported in the study; mild side effects did not differ significantly between the TXA and placebo groups.
Gopinath et al., 2023 [13]	Pragmatic, open-label, parallel-group randomized controlled trial	110 patients (nebulized TXA group: 55, IV TXA group: 55)	Hemoptysis in pulmonary tuberculosis (n=84)	Nebulized TXA 500 mg every eight hours vs. IV TXA 500 mg TID	The nebulized TXA group experienced more cessation of bleeding than the IV TXA group at 30 minutes (72.3% vs. 50.9%, p=0.0019), experienced a significant reduction in hemoptysis volume at all time points through 24 hours, and had fewer patients who required bronchial artery embolization.	Two patients in the nebulized TXA group experienced bronchoconstriction that resolved after administration of a nebulized short-acting beta-agonist.
Kinoshita et al., 2019 [14]	Retrospective nationwide cohort study	28,539 patients (TXA group: 17,049; no-TXA group: 11,490)	Hemoptysis in bronchopulmonary carcinoma (n=4692), cystic fibrosis (n=5517), respiratory infection (n=10109)	IV TXA 2,000 mg or less vs. no TXA	Propensity score matching yielded 9,933 paired patients. In the matched analysis, TXA use was associated with significantly lower in-hospital mortality, shorter hospital stays, and lower healthcare costs.	There was no statistically significant increased risk of thromboembolism or seizure in the TXA group.
Tscheikuna et al., 2002 [17]	Randomized, double-blind, placebo-controlled trial	46 patients; TXA group: 21, placebo group: 25	Hemoptysis stratified by bleeding amount: blood-streaked sputum, <20 mL frank blood, or 20 to 500 mL blood/day	TXA compared with placebo over a one-week study period	TXA did not shorten the duration of hemoptysis compared with placebo; baseline imbalance was noted, with the placebo group tending to have less underlying lung disease and more normal chest X-rays.	Low incidence of side effects reported.
Alkazemi et al., 2023 [18]	Single-center retrospective matched cohort study	72 patients (TXA group: 14, control group: 58)	Hemoptysis in airway disease (n=5), malignancy (n=13)	Nebulized TXA 500 mg once every six hours vs. no TXA	There was no significant difference in the need for invasive intervention (35.7% vs. 56.9%; p=0.344), time to hemoptysis resolution, duration of mechanical ventilation, hemoptysis recurrence, or hospital length of stay.	Nebulized TXA did not result in more documented thromboembolic events or seizures, and no bronchospasms were reported.
Hankerson et al., 2015 [19]	Case report	46-year-old male	Invasive laryngotracheal tumor with a history of stage IV piriform sinus and thyroid squamous cell carcinoma	Nebulized TXA 1000 mg over 30 to 45 minutes	Hemoptysis stopped within 15 minutes of administration.	No significant adverse events were reported in the study.
Agrawal et al., 2025 [20]	Randomized controlled, double-blind trial	56 patients (TXA group: 27; control group: 29)	Hemoptysis with pulmonary cavity (n=33), fibrotic lung lesion (n=23)	Nebulized TXA 500 mg every eight hours for two days vs. placebo (nebulized normal saline)	A higher proportion of TXA-treated patients achieved complete resolution of hemoptysis (63% vs 24%, p=0.003). Nebulized TXA produced greater reductions in hemoptysis frequency and volume on day one compared to the control, with significance maintained on day two.	No serious adverse events occurred. Minor adverse events, including cough and throat irritations, were similar between groups.
Solomonov et al., 2009 [22]	Case series	Six patients	Lung cancer or metastatic thyroid carcinoma (n=3)	Endobronchial TXA 500 mg once (n=2), nebulized TXA 250 to 500 mg, ranging from two to four x/day for two days to three months (n=4)	In all cases, the bleeding stopped with the first dose of TXA	No adverse events reported
Fekri et al., 2017 [23]	Randomized controlled trial	50 patients (TXA group: 25, adrenaline group: 25)	Bronchoscopy with biopsy	Endobronchial TXA 500 mg as needed vs. endobronchial adrenaline 1 mg	There was no significant difference in time to bleeding control between TXA and adrenaline (p=0.908). Only one patient in the TXA group required repeated dosing compared to eight in the adrenaline group.	No adverse effects reported
Márquez-Martín et al., 2012 [24]	Prospective observational study	48 patients	Iatrogenic hemoptysis (n=28) and noniatrogenic hemoptysis (n=20; malignancy (n=11), bronchiectasis (n=10))	Endobronchial TXA 500 mg diluted in 15 mL normal saline for 20 to 30 seconds	In the noniatrogenic group, bleeding control was achieved in 11 patients (39.2%), compared with all patients in the iatrogenic group.	No adverse effects reported
Badovinac et al., 2023 [25]	Single-center, double-blind, randomized controlled trial	130 patients (TXA group: 65, adrenaline group: 65)	Iatrogenic hemoptysis during flexible bronchoscopy	Endobronchial TXA (100 mg, 2 mL) up to three applications vs. endobronchial adrenaline (0.2 mg, 2 mL, 1:10000)	There was no significant difference between adrenaline and TXA in controlling bleeding, which was successfully achieved in 83.1% of patients (54/65) in both groups (p=1.0), with a similar number of applications needed to stop bleeding.	No adverse effects reported
Ghadirzadeh et al., 2025 [26]	Case series	Two patients	Hemoptysis with COPD (n=1), bronchiectasis (n=1)	IV TXA 500 mg every eight hours and nebulized TXA 500 mg every eight hours	In both cases, bleeding stopped after administration of combination TXA.	No adverse effects reported
Caban et al., 2025 [27]	Retrospective bicenter observational study	189 patients	Hemoptysis with bronchopulmonary cancers (n=74), infections (n=52), and iatrogenic causes (n=24)	Nebulized TXA 500 mg/5 mL three times daily over ≥15 minutes	Hemoptysis cessation within five days was achieved in 162 patients (85.7%) with a median hospital stay of seven days. Treatment failure (27 patients) was more frequent in those with cancer (70.4%).	No adverse effects reported
Calvo et al., 2016 [28]	Case series	Four patients	Hemoptysis with lung cancer (n=3), bronchiectasis (n=1)	Nebulized TXA 250 mg every eight hours (n=1), 500 mg every eight hours (n=2), 250 mg every 12 hours for approximately 15 minutes	Bleeding resolved within 48 hours in all cases, with the earliest being six hours.	No systemic adverse effects, bronchospasm in one patient after the third dose, which was managed successfully with bronchodilators.
De Boer et al., 1991 [29]	Case series	Two patients	Hemothorax with malignant mesothelioma	Oral TXA 1 g four times daily, with a single daily intrapleural dose of 5 g administered through the thoracic drain for two days	In the first patient, bleeding stopped within 24 hours but recurred with discontinuation, requiring maintenance therapy. In the second patient, bleeding was controlled with the discontinuation of oral TXA by day eight.	No adverse effects reported
Hunt et al., 2024 [30]	Case report	One patient	Hemoptysis with diffuse alveolar hemorrhage	Adjunctive use of TXA	Demonstrates the use of TXA alongside other hemostasis agents.	No adverse effects reported
Rushlow et al., 2024 [31]	Case report	One patient	Hemoptysis with hemorrhagic pneumonia	Nebulized TXA	Swift resolution of hemoptysis.	No adverse effects reported
Fiore et al., 2024 [32]	Case report	One patient	Hemoptysis with atypical pneumonia	Nebulized TXA	Improvement of hemoptysis.	Small pulmonary thromboembolism managed with alteplase.

*Nonmassive Hemoptysis* 

Multiple randomized and observational studies support the use of TXA in nonmassive hemoptysis across diverse etiologies [6,13,19-21]. In a randomized controlled trial, inhaled TXA achieved bleeding cessation within five days in 96% of patients compared to 50% with a placebo [6]. Subsequent trials have demonstrated reduced bleeding burden and shorter hospital stays, with some evidence suggesting improved outcomes with inhaled compared to intravenous administration [13,20]. More recent observational data and meta-analyses further support its effectiveness while highlighting the need for larger, standardized trials [22,27]. 

Bronchiectasis-Associated Bleeding 

Hemoptysis is a well-known complication of bronchiectasis, ranging from low-volume bleeding to potentially fatal hemorrhage. In a U.S. bronchiectasis research registry, hemoptysis was reported in 23% of patients [33]. Management depends on bleeding severity and may include treatment of infection, bronchoscopic intervention, bronchial artery embolization, or surgical resection. Studies evaluating TXA for hemoptysis frequently include patients with bronchiectasis-associated bleeding as a subgroup within broader cohorts [6,24,26]. In a 2016 case series, nebulized TXA was administered to four patients with hemoptysis due to bronchiectasis or lung metastases, with bleeding cessation reported within 48 hours [28]. In the randomized controlled trial by Wand et al., approximately one-third of the study population had underlying bronchiectasis, a finding also reflected in the study by Tsai et al.; however, subgroup-specific efficacy data remain limited [5,6]. 

*Malignancy-Related Hemoptysis * 

Malignancies that erode into the airways are a well-recognized cause of chronic hemoptysis [1]. While most cases are associated with primary lung cancer, hemoptysis may also occur in metastatic renal cell carcinoma, thyroid malignancies, and malignant mesothelioma. Evidence for TXA use in malignancy‑related hemoptysis is largely derived from case reports and small case series, including early descriptions by De Boer et al. and subsequent reports such as those by Solomonov et al. and Hankerson et al., all of which describe rapid control of bleeding across diverse cancer‑associated etiologies [19,22,29]. 

*Infection-Associated Hemoptysis and Diffuse Alveolar Hemorrhage (Adjunctive Therapy)* 

Respiratory infections, including tuberculosis, acute bronchitis, pneumonia, and aspergillosis, can damage airway mucosa, erode blood vessels, and result in hemoptysis [1,4]. Diffuse alveolar hemorrhage has multiple etiologies, including vasculitis, drug toxicity, rheumatic disease, and, rarely, respiratory infections, with TXA used alongside other therapies for early bleeding control [30]. Multiple case reports describe the use of various TXA routes in hemoptysis, including pneumonia-associated cases in which bleeding resolution was reported [31,32]. In a pilot randomized controlled trial conducted in India by Gopinath et al., tuberculosis and allergic bronchopulmonary aspergillosis were common underlying etiologies among enrolled patients [13]. Although nebulized TXA was associated with favorable short-term bleeding outcomes compared with IV TXA, the findings should be interpreted cautiously because of the pilot design, limited sample size, and single-center setting. Tranexamic acid may serve as a temporizing measure while arranging definitive interventions such as bronchoscopy or bronchial artery embolization for control of hemoptysis [4,13]. 

Discussion 

*Integration Into Hemoptysis Management Algorithms* 

Tranexamic acid may be integrated into hemoptysis management algorithms as an adjunctive or temporizing therapy rather than a replacement for definitive intervention. In patients with nonmassive or localized hemoptysis, inhalational TXA may help reduce bleeding volume, promote short-term bleeding cessation, and decrease the need for invasive procedures in selected patients, although it should not be viewed as equivalent to definitive interventions when bronchoscopy, bronchial artery embolization, or surgery is clinically indicated [6,12,21]. In cases of severe or life-threatening hemoptysis, TXA is best viewed as a bridging therapy to stabilize bleeding while arranging definitive management such as bronchoscopy or bronchial artery embolization [1,2, 5-7,31]. Endobronchial administration during bronchoscopy may provide targeted hemostasis in cases of localized airway bleeding or procedure-related hemorrhage, although supporting evidence remains limited to small trials, pilot studies, and case series [22-25]. Tranexamic acid, including nebulized formulations, has been increasingly discussed in emergency medicine and pulmonary literature as a reasonable adjunctive option in appropriately selected patients, but its role should be interpreted in the context of limited and heterogeneous clinical evidence [16,21]. 

*Limitations and Considerations * 

Despite increasing use of both IV and inhalational TXA, direct comparative evidence remains limited and heterogeneous. Current studies suggest that nebulized TXA may offer theoretical advantages in select cases of nonmassive or localized hemoptysis because it delivers therapy directly to the airway; however, more rapid or targeted bleeding control has not been consistently demonstrated across studies [6,13,21]. Available studies have reported reductions in bleeding volume, bleeding frequency, and need for invasive intervention, but the evidence is not sufficiently robust to establish superiority of nebulized TXA over intravenous therapy [6,13,20,21]. Importantly, not all studies have demonstrated benefit across measured outcomes. Some trials and observational studies reported no significant differences in selected endpoints such as bleeding duration, recurrence, requirement for invasive intervention, or adverse events, underscoring the need to interpret positive findings in the context of heterogeneous populations, variable dosing strategies, and inconsistent outcome definitions [4,12,15,17,18]. At least one comparative trial should be interpreted cautiously because of its pilot design, limited sample size, and single-center setting [13]. Intravenous TXA provides systemic antifibrinolytic activity and may be considered when local delivery is not feasible or when bleeding is diffuse, but hemoptysis-specific data remain limited, and dosing strategies vary across studies [4,12-15]. Interpretation of the overall literature is constrained by small sample sizes, single-center designs, variable definitions of hemoptysis severity, inconsistent dosing regimens, heterogeneous outcome measures, and limited adverse event reporting [4,12,13,21]. Many studies rely on short-term endpoints such as bleeding cessation time and are underpowered to assess clinically meaningful outcomes such as recurrence, mortality, thromboembolic risk, or long-term safety [4,12,21]. As a result, current evidence supports a complementary rather than definitive hierarchy between routes, with inhalational TXA considered most applicable in selected cases of nonmassive or localized hemoptysis and IV therapy reserved for cases in which systemic therapy is clinically appropriate [6,13,21]. 

Knowledge Gaps and Future Directions 

Key areas requiring further investigation include optimal dosing regimens, role in massive hemoptysis, comparative efficacy of inhalational versus IV routes, long-term recurrence prevention, and standardized algorithmic integration. Larger multicenter randomized trials are needed.

## Conclusions

Tranexamic acid is a promising adjunctive therapy in the management of hemoptysis, particularly as part of early stabilization or bridging therapy while definitive evaluation and intervention are arranged. Inhalational TXA may offer localized bleeding control with limited systemic exposure and a favorable safety profile. However, the evidence remains evolving and is largely based on small- to moderate-sized studies with heterogeneous designs.

Definitive procedural interventions remain essential for life-threatening hemoptysis. Current evidence supports TXA as an emerging, increasingly utilized option in selected patients, especially for nonmassive or localized hemoptysis and as a temporizing measure before bronchoscopy or bronchial artery embolization, but larger, standardized trials are needed to better define the optimal route, dosing, safety, and placement within treatment algorithms. 
